# Testing the Hypothesis of Biofilm as a Source for Soft Tissue and Cell-Like Structures Preserved in Dinosaur Bone

**DOI:** 10.1371/journal.pone.0150238

**Published:** 2016-02-29

**Authors:** Mary Higby Schweitzer, Alison E. Moyer, Wenxia Zheng

**Affiliations:** 1 Department of Biological Science, North Carolina State University, Raleigh, North Carolina, United States of America; 2 North Carolina Museum of Natural Sciences, Raleigh, North Carolina, United States of America; College of the Holy Cross, UNITED STATES

## Abstract

Recovery of still-soft tissue structures, including blood vessels and osteocytes, from dinosaur bone after demineralization was reported in 2005 and in subsequent publications. Despite multiple lines of evidence supporting an endogenous source, it was proposed that these structures arose from contamination from biofilm-forming organisms. To test the hypothesis that soft tissue structures result from microbial invasion of the fossil bone, we used two different biofilm-forming microorganisms to inoculate modern bone fragments from which organic components had been removed. We show fundamental morphological, chemical and textural differences between the resultant biofilm structures and those derived from dinosaur bone. The data do not support the hypothesis that biofilm-forming microorganisms are the source of these structures.

## Introduction

Apparent blood vessels, osteocytes, intravascular contents, and fibrous matrix were recovered from demineralized fragments of long bones of *Tyrannosaurus rex* (MOR 1125) [1], and subsequently, from bone of other dinosaurs and other fossil vertebrate remains [2–4]. However, despite evidence from morphological, microstructural, immunological and mass spectrometry data supporting the hypothesis that these structures were endogenous to the dinosaur, an alternative hypothesis was proposed; that these materials were the result of recent invasion of these dinosaur remains by biofilm-forming micro-organisms [5].

In our initial experiments, we considered possible alternative sources for these dinosaur-derived materials. For all studies, we chose dinosaur (MOR 1125) bone fragments from which no visible signs of glues or consolidants were observed. A second dinosaur (MOR 2598) [3] that also produced blood vessel- and osteocyte-like structures was collected without any chemicals, glues or consolidants applied; nevertheless, we tested the hypothesis that glues or consolidants could form such structures. Samples of preservatives commonly applied in the field were prepared, washed in acetone or ethanol, or embedded in the same resin used to section these dinosaur- derived structures. The consolidants dissolved instantly under these conditions, and could not be seen microscopically, with or without staining. The vessels treated in tandem were unaffected [1,3,6–8]. These results do not support consolidants or glues as a source for these vessel structures.

**Fig 1:**
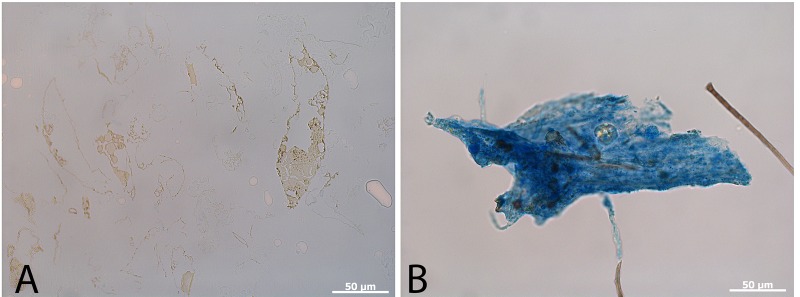
MOR 2598 vessels recovered after demineralization (a) compared with a hyphal mat (b) both stained with the fungal stain cotton blue (see methods). This histochemical stain reacts with fungal components to produce a vivid blue, but dinosaur vessels are unstained.

We eliminated the possibility that the vessels might represent invasion by fungi based upon morphological dissimilarities. Hyphae do not taper after branching and are usually septate [9,10], and the dinosaur vessels do not show these fungal characteristics (Figure 2 in [1]). The vessels were of greater diameter than known fungal hyphae, and like modern blood vessels, they tapered after branching [9], were not septate [9,10], and contained material within them not consistent with spores or other fungal structures (Figure 1e, f in [3]; Figure 2e-g in [11]; Figure 3c, h, n in [2]). Finally, we attempted to stain the structures with a fungi-specific stain, but no reactivity was seen (Fig 1). Thus, a fungal source for the soft tissue dinosaur materials is not supported.

**Fig 2:**
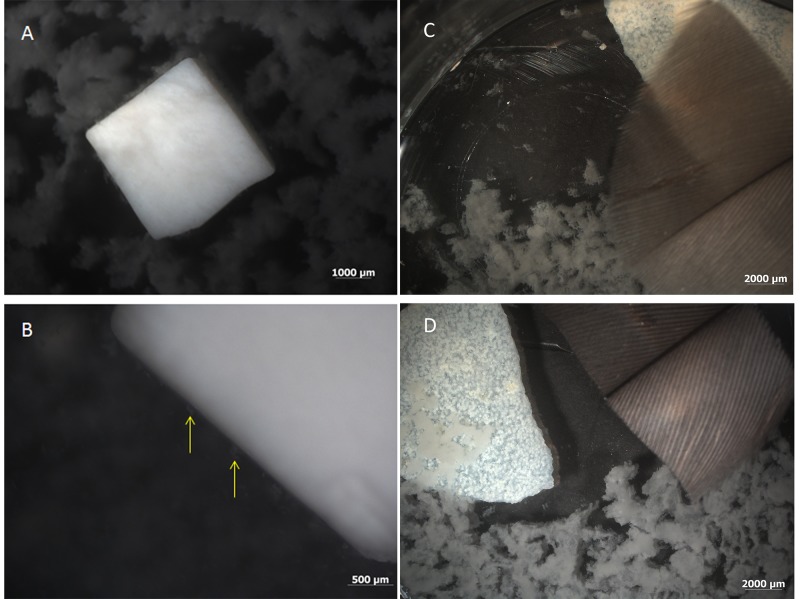
Biofilm growing on cow bone from which organics had been removed (see methods), 48 hours after inoculation. (A) *B*.*cereus* has colonized the bone and can be seen growing on and around the bone at low magnification. (B) *S*.*epidermidis* at higher magnification. Note the interaction of the biofilm with the surface of the bone (yellow arrows). Similar observations were made for both organisms. (C) Feather (tan) and ostrich eggshell (white) in the presence of *B*. *cereus* shows biofilm growth on tissues and in surrounding medium. (D) Feather and eggshell surrounded by biofilm from *S*. *epidermidis*, as above.

**Fig 3:**
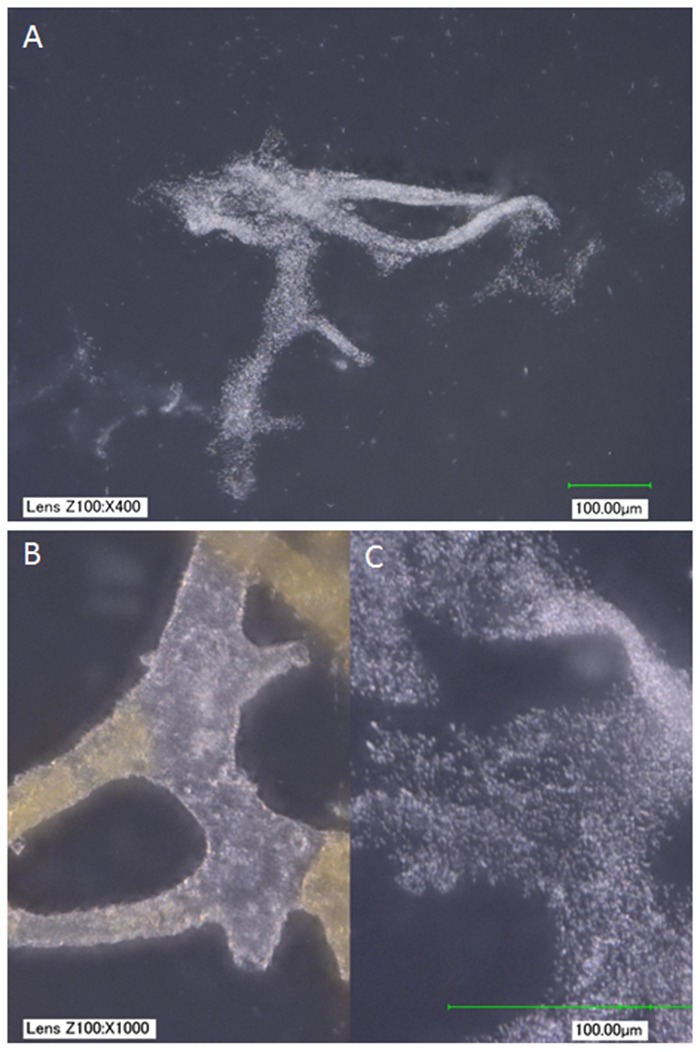
(A) *B*.*cereus* biofilm grown in cow bone from which organics had been removed (see methods). (B, C) Side-by-side comparison of vessels from MOR 2967-C5-1, a second *Brachylophosaurus* specimen from similar deposits (B), and (C) *B*. *cereus* biofilm recovered from cow bone at the same magnification. At low magnification, the biofilm mimics vessel shapes; higher magnification reveals that the biofilm is not hollow, as are the vessels, but rather amorphous clusters of cells. In addition, a red substance is clearly visible and differentially distributed within the hollow dinosaur vessels; no similar features are seen in the biofilm. Images taken with a KEYENCE VHX-2000 digital microscope, scale bar as indicated.

**Fig 4:**
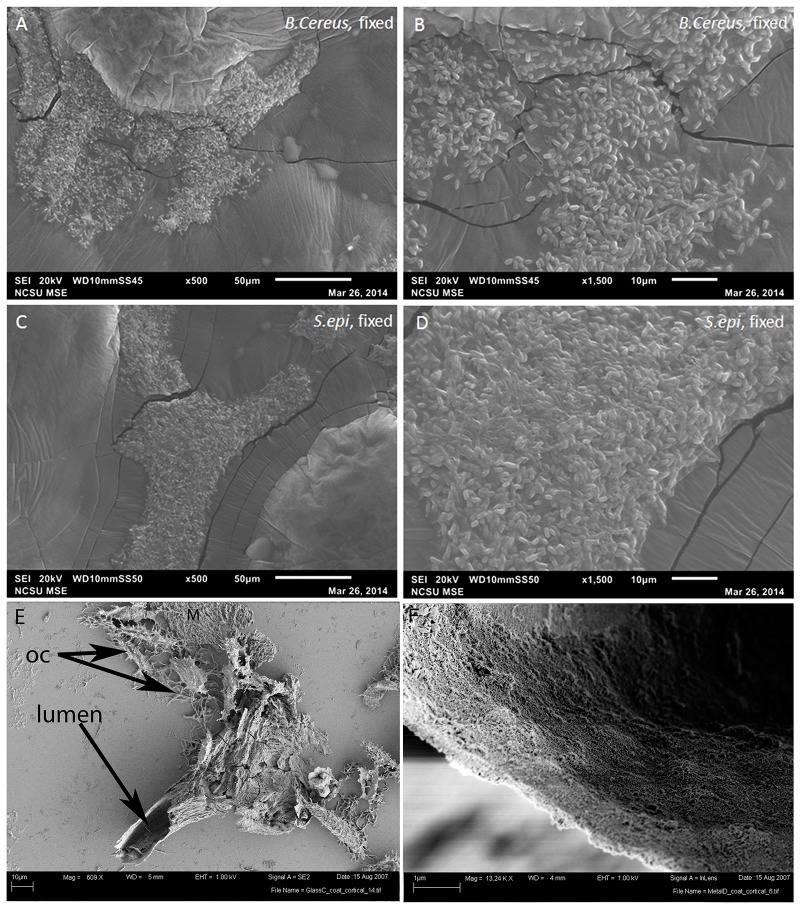
SEM images of (A-B) *B*.*cereus*; (C-D) *S*.*epidermidis*, grown in cow bone from which organics had been removed, and fixed with 10% neutral buffered formalin. At low magnification (A, C) the biofilms are roughly in the shape of the bone channels, but higher magnification demonstrates that they are a rather unorganized mass of cells. These are compared with low (E) and higher magnification (F) of vessels recovered from demineralized dinosaur (MOR 2598) bone. E) Dinosaur-derived vessel, arrows indicate the open vascular lumen and remnant osteocytes (OC) recovered in association with vessels during demineralization. Also associated with the vessel are fragments of fibrous matrix (M). The vessel is hollow, and the lumen continues through the length of the vessel. F) High magnification of vessel wall, showing solid fabric and relatively uniform thickness, neither of which are characteristics of biofilm.

Here, we test the hypothesis that the vessels and/or osteocyte-like structures might arise from microbial invasion by biofilm-forming organisms. Morphologically, the vessels and osteocytes we recovered were not consistent with biofilm. A biofilm is a population of micro-organisms and the exopolymeric substances (EPS) they secrete [12–14], but neither transmission electron microscopy (TEM)[8] nor scanning electron microscopy (SEM)[1,3] revealed distinct microbial bodies (or impressions of these bodies) in association with dinosaur vessels. Additionally, a biofilm may be patchy or uneven in distribution [14–16], with cells detaching and EPS undergoing dissolution once nutrients have been removed [13,17]. Thus, biofilm-forming microorganisms cannot produce the continuous-walled and branching structures of different dimensions that we recovered from fossil bone. Biofilms adhere to substrates, but have no means to maintain shape once that substrate is removed (Fig 2); the dinosaur vessesls maintain a lumen and continuous walls after demineralization of the bone and multiple manipulations (Figs 3 and 4E and 4F). Finally, biofilms are rather amorphous (Fig 2). They may have microscopic internal structure, including pores and channels through which nutrients are exchanged [13,14,18] (and references therein), but they are not morphologically similar to osseous blood vessels.

Furthermore, to our knowledge, modern biofilms have not been *directly* shown to colonize fossil bone, except for one example where fragments of archaeological bone were used as a source to grow biofilms [19]. These bones were relatively recent (thousands of years, vs millions for dinosaurs); thus, it could be expected that they retained organic content, providing a nutrient source for invading microbes. Microbes colonize substrates primarily to obtain nutrients; when the nutrients are no longer available, they detach and the EPS dissipates [17]. Despite recent data to the contrary (e.g. [3,4,6,7]) it is assumed by many that extinct dinosaur bone no longer retains endogenous organics, either DNA or protein (e.g. [20–24]); if devoid of organics, they would not be optimal substrates for microbial growth.

Here, we test the hypotheses that 1) biofilm will colonize and grow within bone from which organics have been removed, as proposed to be the case for dinosaur bone, and 2) biofilm is capable of giving rise to structures similar in morphology to blood vessels. We designed a model system using bovine bone fragments from which organics had been removed, for repeatability and to discount a taxon-specific effect, and to best approximate what is presumed to be the case in dinosaur bone. After organics were removed from modern bone, the remaining bone was either placed in sterile water, or in a medium designed to favor biofilm growth (see methods). We used two different types of microorganisms known to form biofilm, *Bacillus cereus* and *Staphylococcus epidermidis*, to inoculate the bone, again for repeatability and to show that this pattern is not organism-specific (i.e., a property of only one type of biofilm or microorganism). We chose these two microorganisms because both are known to produce biofilm; both are rather common in many environments (e.g. [16,25], and both are readily available. After an incubation period of ~2 weeks, bone fragments were demineralized and remaining biofilm was subjected to both scanning (SEM) and transmission (TEM) electron microscopy. Biofilm was also tested against a range of antibodies (see methods), and reactivity compared with recovered dinosaur vessels exposed to the same antibodies.

## Results

Our first attempt to completely remove organics from relatively large (~2cm x 2cm) pieces of bone was unsuccessful. Even after repeated cycles of extreme heat (~200°C), bleach, and enzyme treatment spanning a period of ~3 months, demineralization of the apatite phase of the bone left visible intact vessels and fibrous matrix. It was important to successfully remove all traces of organics, and thus a nutrient source for microbes, to accurately model conditions within dinosaur bone, because it has been proposed that organics do not persist into the fossil record [20–22]. Intriguingly, if organics persist, microbes *will* invade to obtain nutrients; thus biofilm growth may be independent support for organic preservation in bone. However, even after repeated cycles of harsh treatment, collagen matrix and other components persisted; we could only achieve complete removal of organics after the bone fragments were sufficiently reduced in size (~1cm cubes), and cycles of treatment were significantly prolonged.

Once organics were successfully removed, leaving a bioapatite scaffold to which biofilm-forming organisms could attach, the bone cubes were placed in either sterile water or nutrient broth containing microbes (see methods) and then demineralized to determine 1) if biofilm would form; 2) if this biofilm would retain coherence and morphology of vessels obtained from dinosaur; and 3) if any remnant biofilm would show antibody binding to the same antibodies, and in the same pattern, as observed for vessels recovered from dinosaur bone.

Fig 2A shows that when bone is placed in a nutrient medium, biofilm-producing microbes and biofilm will form, using the bone as a substrate. Both test organisms showed similar results, producing a flocculent biofilm on the surface and in the surrounding medium (Fig 2A and 2B). Higher magnification shows that biofilm is deposited on and interacts with the bone surface (Fig 2B, arrows). However, without the addition of nutrients to facilitate microbial growth, no biofilm was observed. We included feather, a non-biomineralized tissue, and eggshell, biomineralized but not bone, to show that biofilms from both organisms will grow similarly, regardless of tissue type (Fig 2C and 2D), if nutrients are present.

After full colonization of the bone by both organisms in nutrient broth, the mineralized bone scaffold was removed by calcium chelation using ethylenediaminetetraacetic acid (EDTA). Fig 3A shows that the biofilm product superficially resembles blood vessels at low magnification in both chemically fixed and unfixed samples. However, these structures were never observed to possess a lumen and showed complete loss of integrity when manipulated, or even with simple rinsing. Both samples disintegrated (Fig 3C), in contrast to vessel structures recovered from dinosaur bone (Fig 3B), which were intact and retained shape, texture and hollow lumen, even after being manipulated multiple times [1,2].

Scanning electron microscopy (SEM) reveals further differences between the morphology of the bone-grown biofilm and vessels retrieved from dinosaur bone. Because unfixed biofilm samples did not retain their shape, we applied chemical fixatives to biofilm produced by *B*. *cereus* (Fig 4A and 4B) and *S*. *epidermidis* (Fig 4C and 4D) (see methods). These show that while the general shape of vessels is retained in low magnification (Fig 4A–4C), higher magnification images (Fig 4B and 4D) show a rather patchy distribution of microbial bodies, clearly discernible within or associated with an amorphous matrix. The structures are flat and two-dimensional, with no evidence of a lumen, which is consistently present in dinosaur vessels. Fig 4E and 4F show vessels isolated from demineralized bone of a *Brachylophosaur canadensis*, MOR 2598 [3]. These were not treated with fixatives, or any chemicals other than the EDTA used to demineralize the bone, yet the vessels retain shape and integrity and clearly differ in microstructure from the biofilm-produced material. Lower magnification shows branching of a portion of a vessel recovered from this dinosaur bone, with lumen visible at one end (Fig 4E, arrow). Fracturing from exposure of the fragile vessel to high vacuum used to image shows that the vessel is hollow throughout its length. High magnification of another dinosaur vessel shows that the wall of the vessel is smooth and solid, whereas biofilms are not generally uniform in structure, and are patchy in distribution (Fig 4B) [12,18]; their distribution is correlated to nutrient sources. In addition, SEM of dinosaur vessels has never shown what could pass for microbial bodies associated with the soft tissue structures. In fact, in the only work to show biofilm residing in relatively recent human bone [19], SEM imaging presents as a rather foamy appearance, with occasional microbial bodies visible (see [19], Figs 1 and 2), not a coherent structure with discernible shape as observed in these dinosaur materials.

**Fig 5:**
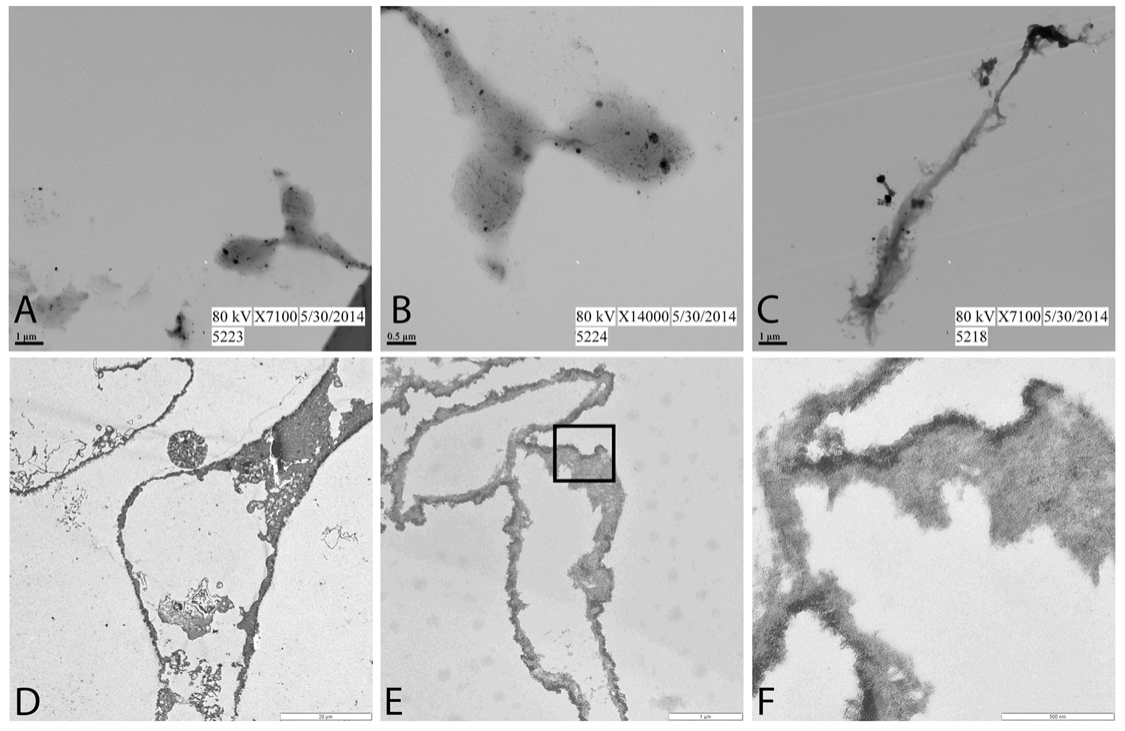
TEM images of biofilm recovered from modern bone from which organics had been removed (A-C), compared with representative vessels recovered from dinosaur bone (D-F). A, low and B, higher magnification of biofilm from bone that has been chemically fixed in 10% formalin. C. *B*. *cereus* biofilm, grown in bone but not chemically fixed (see methods). D. isolated vessel from *Tyrannosaurus rex* (MOR 1125). Vessel is naturally stained, most likely because of the intimate association of iron with these vessels [8]. E, low and F, higher magnification of a separate preparation of dinosaur vessels, showing open lumen and regular vessel wall structure.

Fig 5 compares TEM images of biofilm grown in bone with those of isolated vessels from the *Tyrannosaurus rex* MOR 1125 [1]. Little structure can be seen in biofilm samples, even after staining, and there is no visible lumen. Fig 5A shows *B*. *cereus* biofilm, fixed with neutral buffered formalin before demineralization of inoculated modern bone (methods). Fig 5B shows a higher magnification of this material, while Fig 5C shows biofilm from modern bone that has not been fixed prior to imaging. Fig 5D shows two isolated blood vessel sections from demineralized *Tyrannosaurus rex* (MOR 1125) bone visualized using TEM. The vessel walls are distinct, relatively uniform, and surround a lumen. Fig 5E shows another fragment of vessels, again with distinct wall and visible lumen. Fig 5F is a high magnification view of the material in the box in 5e. Elemental analyses [6,8] show that the vessels are infused with nanoparticles of iron, evidenced by the darker band in Fig 5F.

**Fig 6:**
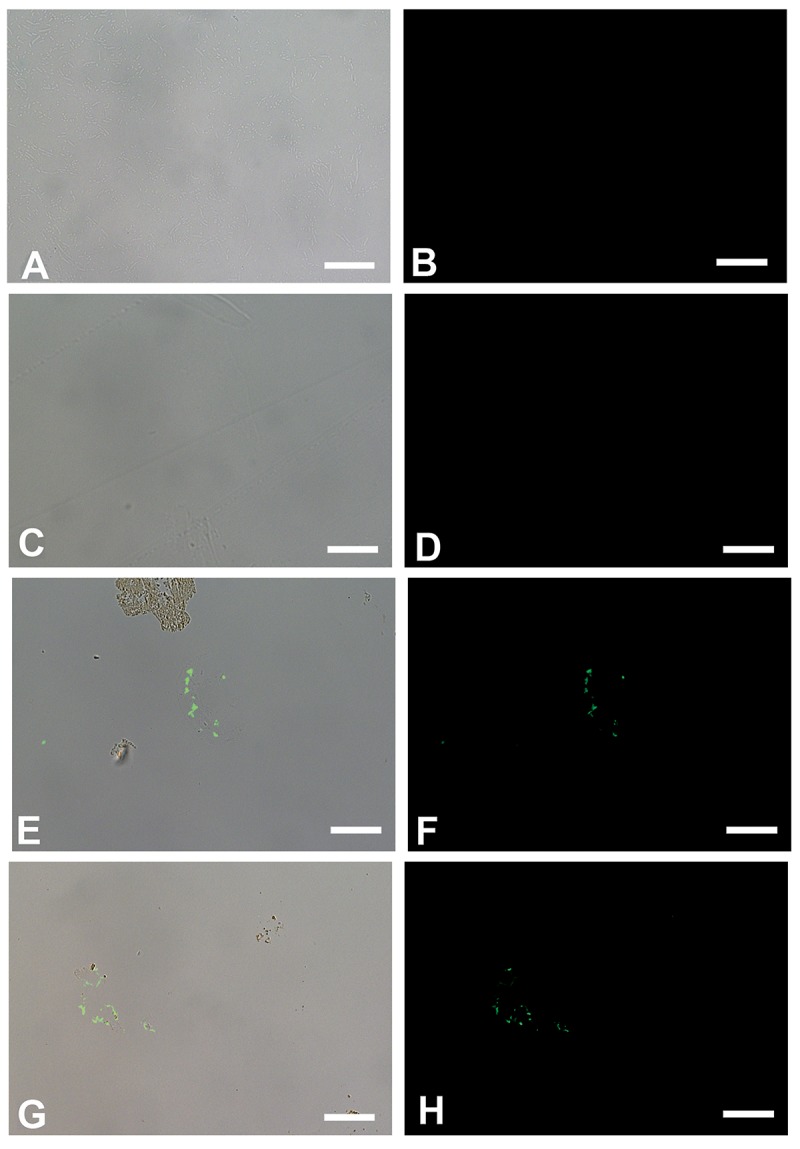
*In situ* immunohistochemistry of biofilms (A-D) and dinosaur vessels (E-H) exposed to polyclonal antibodies raised against actin, a cytoskeletal protein (see methods for details). A, C, E, G are overlay images, showing where on the tissue the antibodies bind; B, D, F, H are fluorescent images showing only antibody binding, as represented by the green fluorescence of the FITC label. A, B represent *B*.*cereus* biofilm grown in bone; C,D is *S*. *epidermidis* biofilm; E, F is vessels from *B*. *canadensis* (MOR 2598 [3]), G, H, vessels from *T*. *rex* (MOR 1125 [1]). Scale bar for all images = 20 μm

**Fig 7:**
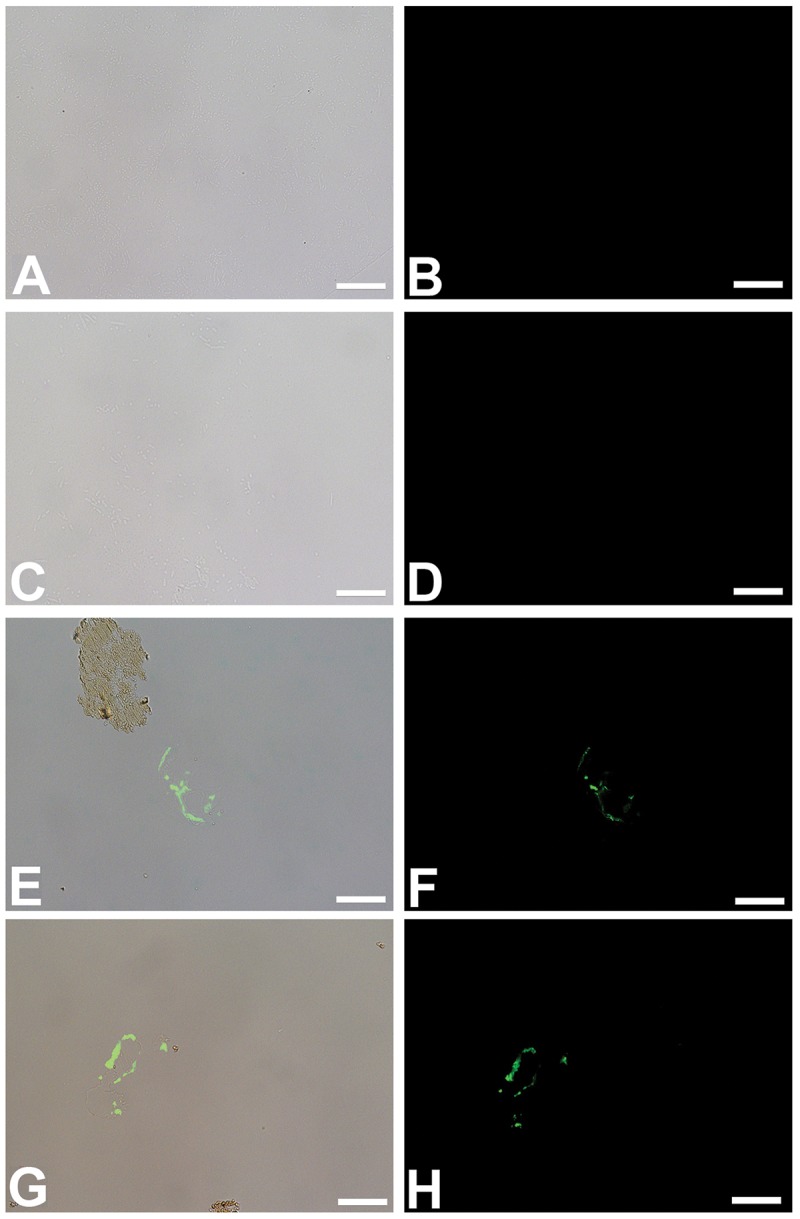
*In situ* immunohistochemistry of biofilms (A-D) and dinosaur vessels (E-H) exposed to polyclonal antibodies raised against elastin, a highly conserved, robust protein found in vascular walls [28,34] (see methods for details). As above, A, C, E, G are overlay images; B, D, F, H are fluorescent images showing only antibody binding, as represented by the green fluorescence of the FITC label. A, B represent *B*.*cereus* biofilm grown in bone; C,D is *S*. *epidermidis* biofilm; E, F is vessels from *B*. *canadensis* (MOR 2598 [3]), G, H, vessels from *T*. *rex* (MOR 1125 [1]). The specificity of these antibodies is illustrated by the binding to the isolated vessel in E, but not the portion of fibrillar matrix that is naturally stained and visible in the upper left. These antibodies do not bind to either biofilm. Scale bar for all images = 20 μm

**Fig 8:**
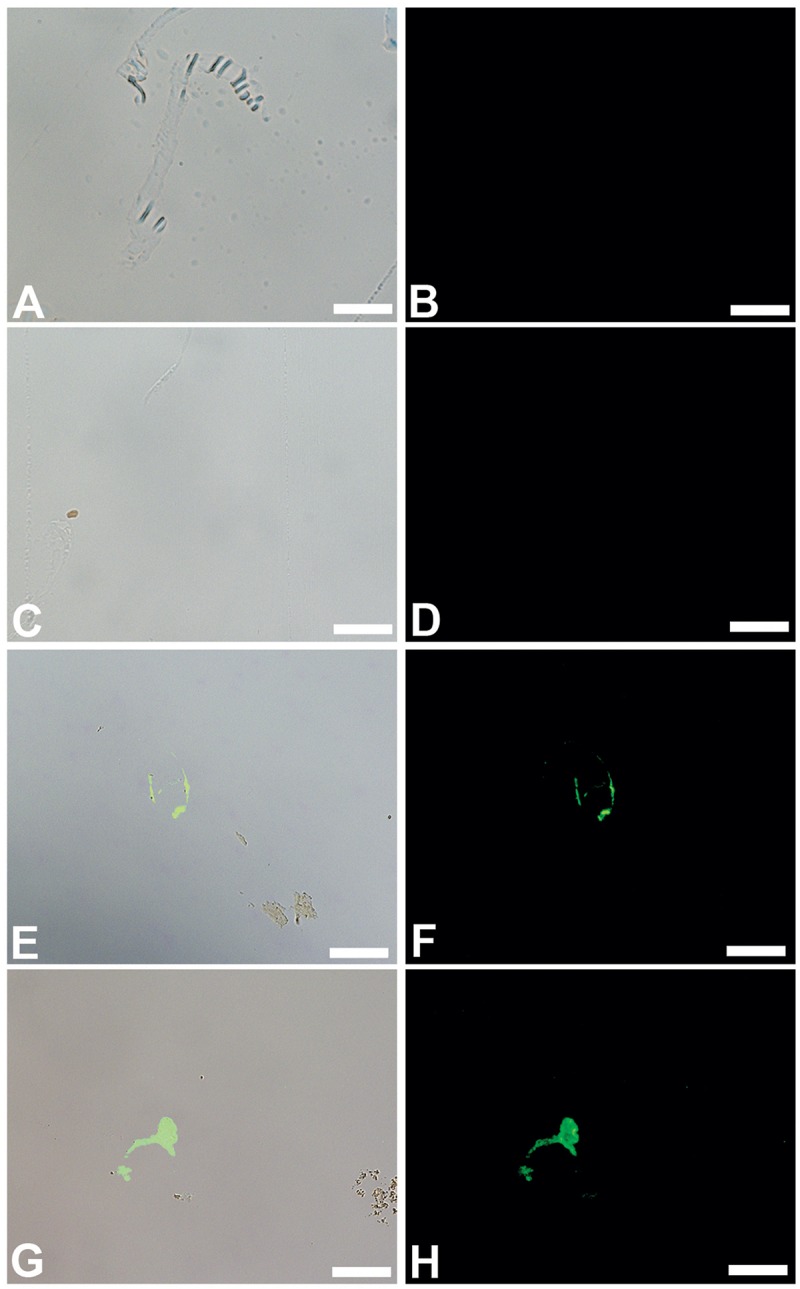
Overlay and fluorescent images of *B*. *cereus* (A, B), *S*. *epidermidis* (C, D) *B*. *canadensis* vessels (E, F) and *T*. *rex* vessels (G, H) exposed to antiserum raised against purified ostrich hemoglobin. No binding of these antibodies to either biofilm is visualized, but specific binding to vessels from both dinosaurs is seen. Scale bar for all images = 20 μm

**Fig 9:**
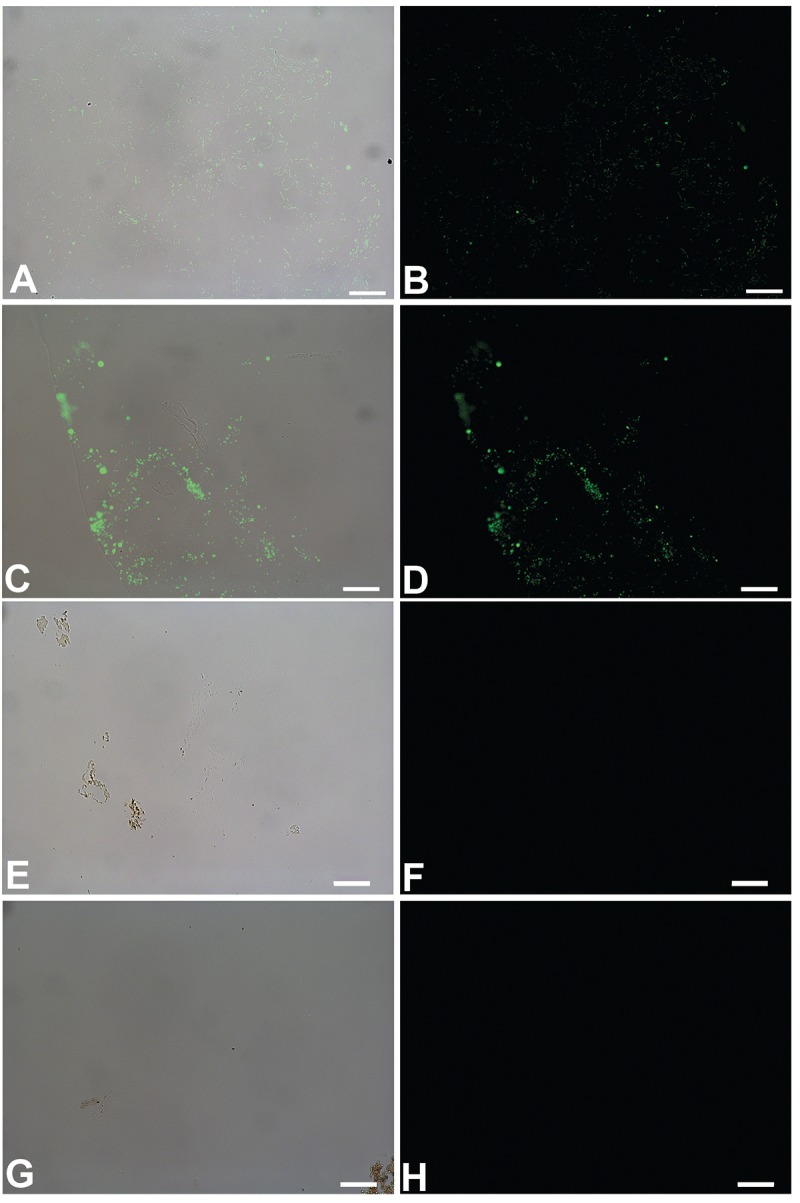
Overlay and fluorescent images of *B cereus* (A, B), *S*. *epidermidis* (C, D) biofilms, *B*. *canadensis* (E, F) and *T*. *rex* (G, H) vessels, exposed to antibodies raised against peptidoglycan, a bacterially produced glycosaminoglycan that is a component of both bacterial cell walls and the EPS they secrete [33]. Binding of these antibodies is evident in both biofilm products, but no binding is seen of these antibodies to either dinosaur vessel. Scale bar for all images = 20 μm

We show that these microbial structures, in both cases, are chemically distinct from dinosaur vessels as well. Antibodies to proteins present in, but not necessarily exclusive to vertebrate vasculature, bind specifically to vessels retrieved from dinosaur bone, but do not bind to biofilms grown in bone. Polyclonal antibodies raised against actin, a eukaryotic protein that forms the cytoskeleton of vertebrate cells [26,27], bind to the vessel walls of both dinosaurs (Fig 6E–6H), but do not react to biofilms prepared in the same manner (Fig 6A–6D). Similarly, antibodies raised against elastin (Fig 7), a highly durable protein found in the walls of virtually all vertebrate blood vessels [28,29], and hemoglobin (Fig 8), the protein that functions to carry oxygen to tissues and the main protein found in red blood cells [30–32] also bind with specific reactivity to the dinosaur vessels (Figs 7E–7H and 8E–8H) but again, do not react with either biofilm when visualized using identical parameters (Figs 7A–7D and 8A–8D). Finally, antibodies to peptidoglycan, a glycosaminoglycan produced exclusively by bacteria [33], bind to the biofilms grown in bone (Fig 9A–9D), but do not bind to the dinosaur vessels under identical treatment conditions (Fig 9E–9H).

## Discussion

The idea that original molecular components can survive across geological time is controversial [22,35,36] (but see [37,38]). The persistence of still-soft and flexible materials like blood vessels and bone cells in dinosaur bone is even more so. It has been predicted that DNA has limited stability [22,39], and will degrade to single bases under best-case conditions in about 6 million years. These time estimates are based upon decreased DNA recovery in a series of Holocene fossil and subfossil materials dating to ~7500 years, then extrapolation of these data to longer time frames [22]. Alternatively, determining breakdown rates of ‘naked’ DNA in solutions has also been used as a proxy for time [39,40]. Verifiable and accepted DNA sequences have yet to be published from bones older than 1Ma.

Protein survival rates vary depending upon the protein tertiary/quaternary structure and burial history, among other factors [24], but in general, are predicted to outlast DNA. Even so, conventional wisdom states that proteins also will not survive into deep time [24]. Therefore, structures derived from proteins, such as the soft tissues we reported, were proposed to be derived from contamination [5], because if constituent molecules cannot persist in geological time, how can the structures they comprise do so?

We argued for endogeneity of these structures based upon the following lines of evidence: 1) Morphology was inconsistent with other alternatives, but consistent with vessels and osteocytes; 2) Textural differences between the structures were maintained at sub-micrometer levels (i.e., fibrous matrix was distinct, both from osteocytes embedded within it [7], and from vessels); 3) Chemical differentiation between adjacent structures was supported by color differences between vessels, osteocytes, osteocyte filopodia and fibrous matrix (e.g. [2,3]); 4) The materials showed differential response to antibodies raised against eukaryotic or vertebrate proteins [3,6,7,11]; and 5) Molecular sequence data supported a vertebrate source [3,41–43]. Here, we present additional data that *disproves* the hypothesis that these structures derive from biofilm, using actualistic experiments and data from the literature characterizing biofilms and their interactions with substrates.

Microbes most certainly were associated with degrading dinosaur tissues, just as surely as they are found in association with *all* degrading organic matter today. Their presence in the sediments surrounding dinosaur skeletal elements, or even their signature within the bone itself, does not preclude endogenous materials from also being present. Biofilms and bacteria coexist with living organisms, both as contributors to pathology (e.g. [44,45]) and to health (e.g. [46] and references therein). But when an organism dies, the body’s defense mechanisms no longer keep these in check, and they proliferate. This, in addition to microbial invasion from the environment, contributes to breakdown and degradation of organics.

**Fig 10:**
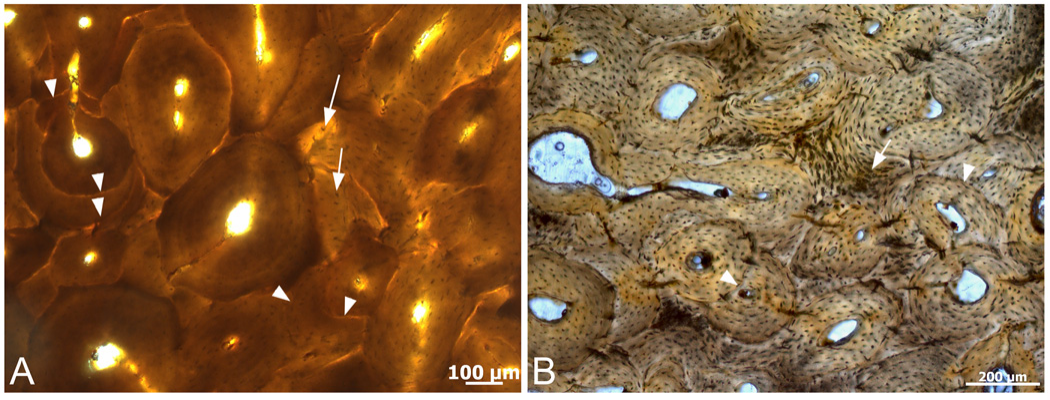
Histological ground sections of A). *T*.*rex* cortical bone and B) *B*. *Canadensis* cortical bone, from which the vessels in this study derive. No evidence of focal destruction Wedl tunneling, or other signs of microbial invasion are visualized. However, histological detail, including multiple generations of secondary osteons (arrowheads) and osteocyte lacunae with cannalicular extensions (arrows) are visible in both samples, attesting to histological integrity. The lack of cement lines (indicating remodeling) in *B*. *canadensis* bone support a younger ontogenetic age for this dinosaur.

However, microbial invasion in bone is easily traced by histological markers, such as Wedl tunneling or focal destruction (e.g. [47,48] and references therein). No such traces were visible in histological sections of dinosaur bone used in this (Fig 10) or other studies. Additionally, although microbial invasion can occur through the vasculature, canaliculi containing osteocyte filopodia are smaller in diameter than single microbes, making transmission via the lacuno-canalicular network difficult ([7] and references therein).

Here, we have shown that it is difficult to fully remove organics from bone while leaving the mineral phase intact, despite harsh treatment. This result was surprising, and has implications for our understanding of the “fossilization” of organic remains. These labile components, when encased within dense cortical bone, may be much more resistant to degradative processes than previously thought.

Because microbes colonize surfaces where organic nutrient sources are available, the removal of organics is important. It is well known and tested that biofilm will employ living tissues as a substrate for growth (e.g. [12,14,16,49]), but here, we test the hypothesis that biofilm will colonize dinosaur bone if no organics remain. To do this, we could either use synthetic apatite, containing no organics, or remove organics from existing bone. We chose the latter, because synthetic apatite is not as porous as bone; therefore it could be argued if biofilm did not grow, that it was not an accurate system to mimic invasion of dinosaur bone.

We show that, if bone pieces are sufficiently small (with correspondingly great surface area), and if they are placed in a nutrient broth containing molecules needed for growth, microbes and their associated biofilm will interact with bone surfaces when organics are removed. Controls of bone with organics removed, then inoculated with microbes, did *not* produce biofilm when placed in sterile water, but required nutrient broth (methods); thus bone alone could not supply these microbes with sufficient nutrients to produce a biofilm. These data have implications for analyses of Cretaceous bone; if modern biofilm can be shown definitively to occupy fossil bone, it may imply retention of original organics as a nutrient source for these microbes.

We have also shown that, if fixatives are applied to the biofilm before removing the bone lattice through demineralization, biofilm can bear superficial resemblance to blood vessels prior to any manipulation. However, unlike vessels, the microbial structures do not possess a lumen, but rather are 2-dimensional casts of the channels in bone. Even when fixatives are applied, these biofilm structures do not hold their shape under manipulation, as do vessels derived from dinosaur bone. This illustrates the need for chemical data, not just morphology, when claiming original microstructural components can be recovered from fossil organisms.

Finally, we show that antibodies raised against eukaryotic proteins not present in microbes bind to dinosaur vessels, but do *not* bind to biofilms grown in bone. Conversely, antibodies raised against microbial peptidoglycan *do* bind to the biofilm grown in bone, but do not bind to dinosaur vessels. These data, together with those put forth in previous publications (e.g. [1–3,6,7,41–43] refute the hypothesis that the vessels, osteocytes, and fibrous matrices derived from dinosaur and other fossil bone tissues are solely derived from microbial invasion. Furthermore, sequence data from chemically extracted dinosaur bone [3,41,42] and isolated dinosaur vessels [50] refutes a microbial source.

It should be noted that microbes do indeed play an important role in the fossilization of organic remains (e.g. [51,52]). To enter the rock record, labile organics or the tissues they comprise must be stabilized before degradation is complete[53]. Although microbes are the main agent of degradation, they have been shown to also play an important role in stabilization by mediating, either passively or actively, rapid mineralization of organic materials [54–58], thus imparting stability. The co-existence of microbes or their products with original organic components has been demonstrated repeatedly ([59,60] and references therein); thus both microbial and vertebrate signal may be detected in fossil studies.

When all data are taken into consideration, an endogenous source for these dinosaur-derived tissues is best supported. Determining the endogeneity of soft tissue dinosaur remains opens new avenues of investigation and supports the hypothesis that original molecules, including proteins and perhaps DNA, may persist in fossil bone. This in turn, may provide alternative ways of testing evolutionary hypotheses. Additionally, understanding the chemical alterations of original organics at the molecular level may have implications for fields as far removed as human health.

## Materials and Methods

Bovine cortical bone (from local Food Lion grocery) were cut into ~2 cm x 2cm fragments (first round). There was no information on the provenance; it is presumed to derive from a local (NC) feedlot. The bone was purchased fresh, but kept frozen until used in this study. Bone was subjected to sequential digestion using 1) 0.5mg/ml proteinase K (PCR grade, Roche 03115879001) solubilized in 30 mM Tris·Cl; 30 mM EDTA; 5% Tween 20; 0.5% Triton X-100; 800 mM GuHCl, pH 8.0 (product information at www.5Prime.com; proteinase K manual_5prime_1044725_032007.pdf) and employed at a working temperature of 50C for 5 days; Trypsin (2 mg/ml, Worthington Biochemical Corp LS003703) solubilized in water with 0.1% calcium chloride, pH 7.8. working temperature 37C [61]; 3) Collagenase A (2mg/ml, Roche 11088785103) solubilized in Dulbucco’s phosphate buffered saline, working temperature 37C) for ~48 hours (At the beginning of each digestion, bone was incubated with enzyme solutions in a vacuum oven at room temperature (RT) for 2–3 hours for better infiltration. After sequential digestions, bone was rinsed in E-Pure water to remove enzyme, baked at 200C for 2 days, then incubated with 10% bleach solution for 3 days). Baking and bleaching steps were repeated two more times to complete one cycle. After repeated sequential treatment for a period of ~3 months, demineralization of bone showed continued presence of vessels, cells and matrix, morphologically unaffected by this treatment. Therefore, this treatment sequence was repeated on smaller blocks of bone (~ 1cm x 1 cm), until demineralization left no trace of organic remains, approximately 8 months. Between bleach and enzyme cycle, bone was washed at least 10 times with E-pure water to remove all chemicals. After all organic material were removed, “naked” bone pieces were washed thoroughly again with e-pure water and air-dried before inoculation with biofilm forming organisms. For handling and treatment of dinosaur vessels please see SOM in references[1–3,6,8].

### Cotton Blue Staining

*B*. *canadensis* (MOR 2598) vessels were embedded for TEM in LR white embedding medium (see below) and 200nm sections were taken and dried onto microscope slides. Modern fungus were not nembedded but dried directly on microscope slides. Both dinosaur vessels sections and fungi were stained using the Lactophenol Cotton Blue Solution protocol from the manufacturer (Fluka Analytical 61335). A drop of Lactophenol Blue Solution was added to each sample, coverslips were applied. Stain was allowed to incubate with both samples for ~ five minutes at room temperature, then samples were imaged using a Zeiss Axioskop 2 plus biological microscope.

### Inoculation and growth of biofilm in organic-free bone

Pieces of bone (organics removed) were inoculated with pure cultures consisting of *Bacillus cereus* (ATCC 14579) or *Staphylococcus epidermidis* (ATCC 49134) in 5% tryptic soy broth (TSB). Pieces of bone were also incubated in sterile E-pure water and sterile E-pure water inoculated with bacteria as negative controls; no biofilm was recovered from these. Setups were maintained in 6-well cell culture plates, rocking at room temperature (~20–22°C) for approximately two weeks. The experiment was halted by fixing the pieces of bone in 10% neutral buffered formalin, washing off excess formalin with E-pure water then demineralizing in 0.5% EDTA (pH = 8.0). The experiment was repeated but without the final fixation step, so that artefact induced by fixation would be recognized.

### Scanning electron microscopy (SEM)

Samples were washed in 3 changes of E-pure water, allowed to air dry mounted on double sided carbon tape, then coated with ~10nm of gold/palladium. Samples were imaged with a JEOL JSM-6010LA analytical SEM controlled by JEOL InTouchScope version 1.05A software.

### Transmission electron microscopy (TEM)

Samples were fixed in 10% neutral buffered formalin, then washed with 1X phosphate buffered saline (PBS) for TEM. Washed samples were dehydrated in 2 changes of 70% ethanol for 30 minutes each, followed by 1 hour incubation in LR white: 70% ethanol (2:1). Samples were then incubated in 3 changes of undiluted LR white for 1 hour each, then embedded in gelatin capsules and polymerized for 24 hours at 60°C. A Leica EMUC6 ultra-microtome with a Diatome 45° knife was used to cut 90nm sections which were mounted on 200 mesh copper grids. Samples were stained with 15% methanolic uranyl acetate (5 minutes), washed in E-pure water, Reynold’s lead citrate (8 minutes) with a final wash. Sections were imaged using Erlangshen ES1000W Model 785 TEM coupled to a CCD 11Megapixel High-speed Digital Camera, and analyzed using Gatan Microscopy Suite (GMS) software.

### Dinosaur materials

Dinosaur bone was provided by the Museum of the Rockies, Bozeman, MT, and include *Tyrannosaurusrex* (MOR 1125) *Brachylophosaurus canadensis* (MOR 2958) and a second *Brachylophosaurus* (MOR341 2967-C5-1). All necessary permits for dinosaur collection were obtained for the described study by the Museum of the Rockies, and complied with all relevant regulations. No additional permits were required for the current study, which complied with all relevant regulations. Specimens are currently reposited at the Museum of the Rockies, Bozeman, MT, USA 59717. These specimens were recovered from federal lands and are publicly reposited, and available to others to study with permission from the curator.

### TEM of MOR 1125 vessels

Vessels were liberated from dinosaur femora cortical bone using EDTA, pH 8.0 (see methods in references [1,2,6]), then either fixed in a solution of 2% paraformaldehyde: 0.01% gluteraldehyde, or left unfixed. Vessels were then rinsed and embedded in 6% agar (Sigma). Samples were rinsed, then dehydrated in ethanol series from 50% to 100%. Some material was post-fixed in 1% OsO4 for 1 hour. Samples were infiltrated with acetone, followed by 1:1 acetone:Spurrs embedding medium, then 100% Spurrs to infiltrate under vacuum overnight. Sections were cut to 60–90 nm and placed on Au 200 mesh EM grids. Some sections were post-stained in uranyl acetate:lead citrate, others were not stained. Sections were imaged using a ZEISS LEO 912 with an acceleration voltage of 100 kV, coupled to a Proscan 2048–2048 digital CCD camera.

### *In situ* Immunohistochemistry (IHC)

Using the same embedded LR white bullets and ultra-microtome used for TEM prep, 200nm sections of either dinosaur vessels, recovered as described, or biofilm, were cut and mounted on 6-well (8mm) Teflon-printed slides and dried overnight at 42°C. Sections were etched with 25 μg/mL Proteinase K in 1X phosphate buffered saline (PBS) buffer at 37˚C for 15 minutes, followed by 0.5 M ethylenediaminetetraacetic acid (EDTA) pH 8.0 (3x10 minutes) and lastly with NaBH_4_ (2x10 minutes). Incubations were separated by two five-minute washes in PBS. Following these steps to accomplish epitope retrieval and quenching of autofluorescence, 4% normal goat serum (NGS) in PBS was applied to occupy non-specific binding sites and prevent spurious binding. Sections were incubated in primary antibody (Polyclonal Rabbit anti- Chicken actin 1:75 (Capralogics, Inc. P00851), polyclonal Rabbit anti-Bovine elastin 1:75 (Courtesy of R. Mecham), Polyclonal Rabbit anti-ostrich Hb 1:200 (GenScript 70594), Monoclonal Mouse anti- peptidoglycan 1:75 (AbD Serotec, 7263–1006)) in primary dilution buffer overnight at 4°C. All sections were then incubated with secondary antibody (biotinylated goat anti-rabbit IgG(H+L) (Vector BA-1000) diluted 1:500 for rabbit primary antibodies, biotinylated goat anti-mouse IgG (H+L) (Vector BA-9200), diluted 1:500 for monoclonal mouse anti-peptidoglycan, for 2 hours at room temperature. Sections were then incubated Fluorescein Avidin D (FITC, Vector Laboratories A-2001) for 1hr at RT. All incubations were separated by sequential washes (2 times for 10 minutes each) in PBS w/Tween 20 followed by two 10minute rinses in PBS. Finally, sections were mounted with Vectashield H-1000 mounting media, and coverslips applied. Sections were examined with Zeiss Axioskop 2 plus biological microscope and captured using an AxioCam MRc 5(Zeiss) with 10x ocular magnification on the Axioskop 2 plus in the Axiovision software package (version 4.7.0.0).

### Dinosaur bone petrographic section

Dinosaur bone was examined for microstructural alteration, including evidence of Wedl tunneling or focal destruction[62]. Cortical bone fragments were embedded in Silmar resin, cut, and ground following previous protocols[63] for histological analyses. Transmitted light images were taken on a Zeiss AxioSkop 2 and cross-polarized images were taken on a Zeiss Axioskop 40.
